# Recent Applications of Capillary Electrophoresis in the Determination of Active Compounds in Medicinal Plants and Pharmaceutical Formulations

**DOI:** 10.3390/molecules26144141

**Published:** 2021-07-07

**Authors:** Marcin Gackowski, Anna Przybylska, Stefan Kruszewski, Marcin Koba, Katarzyna Mądra-Gackowska, Artur Bogacz

**Affiliations:** 1 Department of Toxicology and Bromatology, Faculty of Pharmacy, L. Rydygier Collegium Medicum in Bydgoszcz, Nicolaus Copernicus University in Torun, A. Jurasza 2 Street, PL–85089 Bydgoszcz, Poland; aniacm@cm.umk.pl (A.P.); kobamar@cm.umk.pl (M.K.); 2 Biophysics Department, Faculty of Pharmacy, L. Rydygier Collegium Medicum in Bydgoszcz, Nicolaus Copernicus University in Torun, Jagiellońska 13 Street, PL–85067 Bydgoszcz, Poland; skrusz@cm.umk.pl; 3 Department of Geriatrics, Faculty of Health Sciences, L. Rydygier Collegium Medicum in Bydgoszcz, Nicolaus Copernicus University in Torun, Skłodowskiej Curie 9 Street, PL–85094 Bydgoszcz, Poland; katarzyna.madra@cm.umk.pl; 4 Department of Otolaryngology and Oncology, Faculty of Medicine, L. Rydygier Collegium Medicum in Bydgoszcz, Nicolaus Copernicus University in Torun, Skłodowskiej Curie 9 Street, PL–85094 Bydgoszcz, Poland; arturbogacz@cm.umk.pl

**Keywords:** capillary electrophoresis, herbal drugs, medicinal plants, quality control, quantitative analysis, pharmaceutical analysis

## Abstract

The present review summarizes scientific reports from between 2010 and 2019 on the use of capillary electrophoresis to quantify active constituents (i.e., phenolic compounds, coumarins, protoberberines, curcuminoids, iridoid glycosides, alkaloids, triterpene acids) in medicinal plants and herbal formulations. The present literature review is founded on PRISMA guidelines and selection criteria were formulated on the basis of PICOS (Population, Intervention, Comparison, Outcome, Study type). The scrutiny reveals capillary electrophoresis with ultraviolet detection as the most frequently used capillary electromigration technique for the selective separation and quantification of bioactive compounds. For the purpose of improvement of resolution and sensitivity, other detection methods are used (including mass spectrometry), modifiers to the background electrolyte are introduced and different extraction as well as pre-concentration techniques are employed. In conclusion, capillary electrophoresis is a powerful tool and for given applications it is comparable to high performance liquid chromatography. Short time of execution, high efficiency, versatility in separation modes and low consumption of solvents and sample make capillary electrophoresis an attractive and eco-friendly alternative to more expensive methods for the quality control of drugs or raw plant material without any relevant decrease in sensitivity.

## 1. Introduction

From early times, people recognized plants for their therapeutic properties. Herbal medicine has been used in the management of many ailments for thousands of years and is the oldest method of healthcare in history [1]. Former herbal “drugs” were discovered by trial and error on human subjects; owing to this, the rich tradition of herbalism contains invaluable biomedical information that is continuously being uncovered by contemporary scientists. In contrast, the dynamic progress of drug production in the 20th century was grounded on the isolation of an active substance with a well-defined mechanism of action. Traditional phytochemistry and pharmaceutical chemistry lines led to the development of many safe and effective drugs used in the clinic today for the treatment of, e.g., diabetes, autoimmune diseases, degenerative disorders and cancer. A well-known example of herbalism is traditional Chinese medicine (TCM), which has its followers and is still practiced around the world. Moreover, its potency in numerous illnesses is proved by clinical researchers [2,3]. The use of herbal drugs has been gaining public interest and acceptance. On the one hand, due to poverty and limited access to healthcare in developing countries, people use herbal drugs as a first line of treatment. On the other hand, the most important reason for using herbal therapies in the West is that people believe that herbs will help us live healthier lives and are generally safe for consumption. Individuals often use over-the-counter herbal medicines without medical consultation as home remedies and spend billions of dollars on them. The growing market entails not only abuse but also the adulteration of medicinal products, which can lead to serious health consequences [4]. All drugs, whether of a plant or synthetic origin, should meet safety requirements and be effective [5,6]. The content of bioactive constituents is one of the key parameters in assessing the quality of a herbal medicine. In terms of adulteration, which is a very common problem that is mainly linked to the lack of officially established regulations by governmental organizations regarding the control of herbal preparations marketed as dietary supplements, more effective ways are needed to improve control at the production and marketing stages [7,8,9]. In this light, rapid, simple, accurate qualitative and quantitative methods are essential to evaluate whether herbal formulations fulfill pharmacopoeial requirements. 

Quality control of herbal medicines is a real challenge for analysts because of the complex matrix and several characteristic compounds which must be identified and quantified. Apart from this, an elaboration of the analytic method must be completed and a validation protocol fulfilled. Thus, sophisticated, laborious techniques should be employed, such as high-performance liquid chromatography (HPLC), gas chromatography (GC), high performance thin layer chromatography (HPTLC) and capillary electrophoresis (CE). Among the abovementioned techniques, HPLC is the most prevalent one. Liquid chromatography with a diode-array detector (DAD) in conjunction with a mass spectrometer (MS) is an efficient measure to analyze both known and unknown compounds in a complex matrix [10]. HPLC is also the most frequently used technique for the determination of active constituents in TCMs [11].

Capillary electrophoresis has been commonly applied in the analysis of food, environmental monitoring, clinical diagnostics and pharmaceutical analysis. The latter application has become increasingly popular in recent years due to its high separation efficiency, minimal consumption of required solvents and its small sample volume, low running cost, reproducibility, and versatility in separation modes, making it an attractive, eco-friendly and powerful tool suitable for drug control purposes. Thus, CE has found its place in official pharmacopoeias and pharmaceutical control regulations [12,13]. What is more, researchers have found many solutions to handle the unwanted phenomenon, that is the limitation of poor concentration sensitivity [14]. Since the early 1980s, after Jorgenson and Lukacs demonstrated that the effectiveness of the electrophoresis may be increased if it took place in open-tubular glass capillaries with a diameter of ~75 µm, CE has developed into a flexible and versatile technique, which make it a very attractive alternative to other chromatographic techniques [15,16]. 

**Scheme 1:**
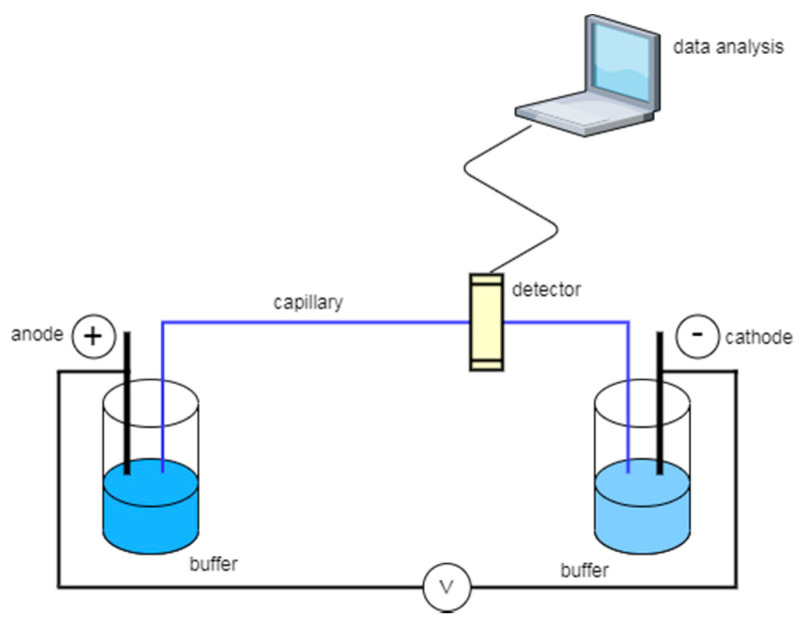
Capillary electrophoresis system.

In CE, analytes are separated in a capillary column with electroosmotic flow (EOF) as the driving force for bulk fluid movement and the action of the electric field. CE requires only simple instrumentation, consisting of a high voltage power supply, two buffer reservoirs, a sample introduction system, a capillary tube, a detector and an output device. See Scheme 1. The capillary is flooded with a solution of background electrolyte (BGE) at a specific pH, which is usually a buffer able to selectively influence the effective mobility. Different capillaries (fused silica or quartz) with internal diameters ranging from 25 to 100 μm and a length of 20 to 100 cm also affect the analysis conditions. Capillaries are placed together with electrodes in reservoirs flooded with the same buffer. In electrophoresis, a mixture of different substances in solution is introduced, usually as a relatively narrow zone, into the separating system, and is induced to move under the influence of an applied potential. The basis for the separation of analytes is in the differences in the electrophoretic mobility of ions as a consequence of the variance in the size and shape of charged particles. Under the influence of an applied electric field, the diverse substances migrate at different rates; thus, after some time, the mixture separates into spatially discrete zones of individual substances [15,17,18]. The majority of capillaries for CE are fabricated from fused silica with characteristic silalol groups on the surface. Those groups dissociate, forming a negative charge in the inner surface of the capillary, attracting a positive charge from the buffer, and finally leads to the formation of an electric double layer. The dispersed cation layer (and its hydration sphere) adjacent to the silica surface tends to migrate towards the cathode, resulting in concomitant fluid migration through the capillary. Anions and cations are separated by electrophoretic migration and eloctroosmotic flow, while neutral species only coelute with the EOF [11,15]. In the terms of expanding sensitivity, introducing different additives such as methanol or acetonitrile is a common phenomenon. Those compounds work by altering viscosity and the polarity of the running buffer, which, in consequence, affects EOF and the electrophoretic mobility of the analyte [15]. As for improvements to the resolution of different compounds, cyclodextrines, for instance, are added to BGE. The use of an appropriate system for the detection of tested substances makes it possible to record the results of the analyses in the form of an electrophoregram [11,18,19].

Over the last few decades, capillary electrophoresis has attracted attention, because the combination of both chromatographic and electrophoretic mechanisms of migration permits the adoption of different separation formats suited to the chemical structure of the analyzed compounds. The following techniques of CE are distinguished: capillary zone electrophoresis (CZE), non-aqueous CE (NACE), micellar electrokinetic chromatography (MEKC), capillary electrochromatography (CEC), capillary isotachophoresis (CITP), capillary isoelectric focusing (CIEF), chiral CE (CCE), capillary gel electrophoresis (CGE), and microemulsion electrokinetic capillary chromatography (MEEKC) [17]. To one the bases of charge density, size, hydrophobicity and chirality, analysts can employ CE to different categories of chemicals [15]. 

The following review summarizes the utilization of CE for the quantification of active constituents in medicinal plants and commercial herbal products, covering the most important applications between 2010 and 2019 (publications in English only). This scrutiny discusses in detail selected physical and chemical (type of buffer, pH) parameters of CE essential for the selective separation of bioactive constituents. Moreover, there is a greater focus on the influence of different pre-concentration and extraction techniques and additives to the background electrolyte for the improvement of resolution and sensitivity. Special emphasis is placed if the reported methods were applied to real samples (medicinal plants, commercial products) and if they were validated. Apart from reporting the current applications of CE, this paper indicates prospects for the further application of this technique.

## 2. Results

### 2.1. Literature Analysis

In the first search in the PubMed and Web of Science database, 682 records potentially meeting the inclusion criteria were found, 363 and 319, respectively. Then, after reviewing the bibliography, duplicates were removed (*n* = 103) and the selected articles were subjected to a subsequent verification by the co-authors. After this, the articles were selected based on the title and abstract. Subsequently, papers describing the application of different methods to CE, CE used only for qualitative information or using CE technique to the analysis of bioactive constituents present in garden and ornamental plants, vegetables and fruits, edible products, beverages, human plasma, blood serum and urine, were eliminated. There were 466 abstracts and papers that were not qualified for this review. One hundred and thirteen articles found in the PubMed and Web of Science databases were used to review the analysis of various bioactive compounds using CE in medicinal plants and herbal drugs.

### 2.2. Capillary Zone Electrophoresis 

The largest number of reported methods for the determination of secondary metabolites in plant material and active substances in herbal medicines recorded in the current systematic review is based on the technique of capillary zone electrophoresis with UV detection. Although the fact that most analytes were determined by molecular absorption, other detection methods, such as fluorometric or electrochemical methods (conductometry, amperometry and potentiometry), were also applied (See Table 1). 

In order to obtain a satisfactory separation and quantification of analytes, it is essential to optimize several parameters such as type of capillary, pH, voltage, injection mode, buffer composition and concentration, additives (type and concentration), etc. This scrutiny reveals that the most suitable BGE to achieve good separation and quantification of different analytes in CZE is borate buffer. 

#### 2.2.1. Separation in CZE

In some cases, adequate separation or quantification with borate buffer as a background electrolyte may be difficult, especially in plant extracts rich in different secondary metabolites or herbal preparations containing many herbs. In this case, the supplementation of the running buffer with some modifiers is a simple and effective way to improve separation efficiency in CE. The positive influence of organic solvents as BGE modifiers on the quality of the separation is expressed as a number of completely resolved peaks. Honegr and Pospíšilova evaluated the influence of methanol, acetonitrile, 2–propanol, and a mixture of 2–propanol and acetonitrile [20]. Liang et al. proved that the addition of β–cyclodextrin (β–CD) and methanol significantly improved the resolution of eight lignans in *Forsythia suspensa*. Excellent separation was accomplished within 15 min with borate buffer as BGE with the addition of 2 mM β–CD and 5% methanol (*v*/*v*) at the voltage of 20 kV, temperature of 35 °C and detection wavelength of 234 nm [21]. An increasingly common approach to increase the resolution between racemic natural products is the addition of cyclodextrins to a running buffer, such as chiral selectors. In addition, microchips are also becoming a popular strategy [22]. In the determination of arecoline by Xiang et al., the additive IL, 1–butyl–3–methylimidazolium tetrafluoroborate (BMImBF_4_) was responsible not only for improvement of separation selectivity but also in the detection sensitivity of the analyte. This additive made the resistance of the separation buffer much lower than that of the sample solution, which resulted in an enhanced field-amplified electrokinetic injection CE [23]. 

#### 2.2.2. Detection Sensitivity

The sensitivity of CE methods is limited by the use of conventional on-line UV detection, which as can be seen in Table 1, is the most common. The path length is rather short due to the capillary diameter, which has a negative influence on the detection sensitivity. This negative phenomenon is usually compensated by the high efficiency and by using low UV wavelengths, but there are also some other ways to overcome this problem. For instance, Song et al. elaborated a CZE method for the determination of aconite alkaloids, where they dissolved the extracts in acetonitrile; in this way they decreased the conductivities of sample solutions. Besides, they used an electro-injection mode which led to a significant improvement in detection sensitivity due to a field-amplified sample stacking effect and values of LOD/LOQ were expressed in nanograms per milliliter [24]. In comparison, the average values of LOQ within the majority of reported studies are in the μg mL^−1^ level (See Table 1, Table 2, Table 3, Table 4 and Table 5). For the analysis of inorganic and organic compounds (together with medicinal products) in an acidic or basic form, contactless conductivity detection can be implemented to overcome the limitations of optical detectors with low sensitivities. This method of detection can be comparable with CE–UV in some applications [7]. At present, the use of electrochemical detection is restricted mainly to conductivity detection, which is mainly employed for compounds that are difficult to detect by UV absorption. Moreover, the use of potentiometric and amperometric detectors is relatively rare [25]. A low limit of detection (LOD) may be also achieved because of the high sensitivity rendered by laser-induced fluorescence (LIF) detection. Reported studies with applications of CE–LIF are characterized by a limit of detection/quantification at the ng mL^−1^ level) for more information, see Table 1). The main disadvantage of fluorescence detection is its necessity for derivatization of the analyte [25]. A microfluidic approach overcomes such inconveniences as poor resolution and poor LOD or LOQ values which herein are reported as microchip capillary electrophoresis coupled with the laser-induced fluorescence (MCE–LIF). This method is characterized by a very small sample and solvent consumption, a short operating time and a high mass sensitivity, which makes it favorable for the determination of minor compounds with fluorescence in complex samples. In a reported paper by Xiao at al., a developed and carefully applied MCE–LIF method for the fast quantification of aloin A and B present in seven aloe plant species and pharmaceutical formulations was presented. In this instance, the LOQ is expressed in ng mL^−1^ [22]. Table 1 shows that in many cases, UV detectors are sufficient for the analysis of active constituents in pharmaceutical formulation or herbal raw material. However, when it comes to analysis of trace analytes in a complex biological matrix, an introduction of extremely sensitive detectors, such as mass spectrometry or laser-induced fluorescence is recommended [26]. 

Some reports describe the fabrication and subsequent application of novel detection electrodes for determining the bioactive ingredients by CE, for instance in *Belamcandae rhizome* [27], in *Bergeniae rhizoma* [28] and in *Cacumen platycladi* [29]. In those cases, the values of LOD/LOQ were also as low as ng per mL^−1^. This approach gives better sensitivity, a considerably lower operating potential, an agreeable resistance to surface fouling, lower operating costs and enhanced stability. Not only does amperometric detection give impressive results, but also, a combination of high separation power of capillary electrophoresis with a high sensitivity of chemiluminescence is becoming very desirable. Wang et al. achieved ultrasensitive determination of epicatechin, rutin, and quercetin by CE with chemiluminescence detection with limits of detection expressed even in pg mL^−1^ [30]. 

#### 2.2.3. Sample Pretreatment Techniques in CZE

Despite its numerous advantages, CE is still considered a niche technique in separation sciences and the use of CE may be limited due to low sensitivity, which is on account of its short optical path and the small capillary dimensions as well as its small sample volume. To remove this inconvenience, sample pretreatment techniques are introduced to the CE system in order to achieve a lower LOD for many analytes, shorten the analysis time, reduce sample consumption, and decrease overall analysis cost. Sample pretreatment is essential for complex matrices, and especially for biological samples. Sample pretreatment may be either attached to CE through a dedicated interface (in-line mode) or online, i.e., unified with the CE separation space during or after sample injection. Liquid phase microextraction and solid phase microextraction are most frequently used as pretreatment techniques prior to sample injection. Among electrophoretic preconcentration techniques during/after sample injection one can distinguish: field-amplified/enhanced sample stacking, large volume sample stacking, field amplified/enhanced sample injection, sweeping, micelle to solvent stacking, isotachophoresis, transient isotachophoresis, and more [26]. 

For the utilization of pre-concentration techniques, Deng et al. elaborated a rapid and simple CE method for the separation and determination of two alkaloids in *Ephedra* herbs. They used a background electrolyte composed of 80 mM of NaH_2_PO_4_ (pH 3.0) with an addition of 15 mM of β–cyclodextrin and 0.3% of hydroxypropyl methyl–cellulose. In this study, the authors took advantage of the field-amplified sample injection and, in the presence of a low conductivity solvent plug, they achieved an approximately 1000-fold improvement in detection sensitivity in comparison to conventional sample injection without any negative impact on resolution [31]. On the other hand, Honegr et al. used large volume sample stacking with polarity switching in order to enhance sensitivity. In this study, sample injection represented 50% of capillary volume and polarity was switched at 1.6 min of analysis, under optimized conditions an average 90-fold heightening of absorbance signal of the analytes was accomplished [32]. The abovementioned authors, Honegr and Pospíšilova, also found a method for the determination of phenolic acids in plant extracts using capillary zone electrophoresis with on-line transient isotachophoretic preconcentration (tITP). The application of preconcentration techniques in this case enabled the injection of large plugs of low concentration samples without overloading the column capacity of the electrophoretic system and consequently led to low detection limits without any decline in separation efficiency [20].

The implementation of extraction techniques prior to separation and detection by capillary electrophoresis is the right approach to obtain an exceedingly sensitive determination. In Table 1 one can find that Zhang et al. employed solid-phase microextraction (SPME) for CE determination of three protoberberines. This group of researchers fabricated a pipette-based device for their new imprinted monolith-based SPME–CE method, which was used for loading, subsequent extraction and final elution of a sample. The positive influence of the addition of methanol to BGE on separation was also noticed. The study confirmed that three protoberberines can be well enriched by the use of imprinted SPME. The limits of detection obtained were lower than in previously reported methods, i.e., 0.1 μg mL^−1^ [32]. Wang et al. described the application of subcritical water extraction (SWE) for the determination of alkaloids in *Sophora*. This relatively new extraction technique was beneficial in terms of operation time, efficiency and lack of organic solvent consumption. Moreover, the electro-injection boosted reproducibility in capillary electrophoresis with field-amplified stacking through the addition of acid to the sample [33]. 

#### 2.2.4. Time of Analysis

An important issue for CE methods and in general for the establishment of a drug quality control method is analysis time. Literature analysis shows that it can be reduced even to 4 min. This impressively short time of analysis is reported by Du and Wang, who applied CE for the determination of berberine in herbal medications [34]. 

#### 2.2.5. CE or HPLC?

Analytical methods elaborated for quality control of herbal preparations based on CE techniques may be an attractive alternative, because of the short analysis time, good separation efficiency, minimal sample, and solvent requirements. However, there is a question of whether CE is able to give comparable results with high performance liquid chromatography (HPLC). In some cases, one can find an interesting answer, for instance, there was no significant difference between the two methods established by Chen et al. using HPLC and CE to determine nine marker components in “samgiumgagambang” (SGMX, herbal medicinal preparation containing 14 herbs) on the basis of the results for the five main constituents in SGMX. What is more, CE stood out for its shorter time of operation (14 min vs. 50 min) and its higher separation efficiency [10]. Dresler et al. in turn verified that capillary electrophoresis may be an alternative to HPLC for assessing the content of metabolites in *Hypericum perforatum* and *H. annulatum* and likewise non-significant differences between those two elaborated methods were found (the difference less than 10%). However, a comparison between LOD and LOQ values achieved with each method demonstrated the advantage of HPLC over CE with respect to detection sensitivity, but the observed difference between these methods can be significant in the analysis of dilute samples with very small amounts of components [35]. Gufler et al. presented capillary electrophoresis as a rapid and potent technique for the analysis of *Urecola rosea* leaf extracts. On the one hand, in terms of qualitative and validation parameters, it was equivalent to HPLC. On the other hand, with respect to operation time and environmental sustainability, CE is definitely beneficial and may be an attractive alternative to HPLC [36]. Table 1 confirms the reader’s opinion that low concentration sensitivity remains a challenge and is the subject of the continuous development of capillary electrophoresis.

#### 2.2.6. Interactions between Analytes and Additives to the BGE

CZE separation is based on the differences between the electrophoretic mobilities of separated compounds. The development of the technique in the form of affinity electrophoresis allows us to obtain highly specific separation through the use of specific ligands (for instance, selective antibodies, proteins, metal ions, or lectins). It should be highlighted that specific interactions between analytes and ligands affect mobility; moreover, they provide possibilities for the isolation and detection of analytes from complex matrices [37]. It is well-established that two main factors influence the electrophoretic mobility, namely the intrinsic physical characteristics of the analyzed compound and chemical additives in BGE interacting with the analyte [38]. Secondary equilibria resulting from additive–analyte interactions are essential to accomplish good resolution. Despite the fact that borate buffer was the most frequently used as a background electrolyte in reported works, various organic solvents and compounds were also added to the BGE to optimize the separations; for instance, surfactants, neutral salts, organic amines, organic salts, and chiral selectors (see Table 1). On the one hand, those additives obviously have an impact on mobility. On the other hand, Table 1 reveals that additives and organic solvents, especially when they go hand in hand with sample pretreatment/preconcentration, have an influence on the detection performance, even when a conventional UV detection is used. Organic solvents and additives are totally different from water, but also from one another in terms of physical and chemical properties. Different solvent properties (in particular pH) strongly influence the acid-base behavior and generally increase the pKa values of analytes (significantly different in organic-water mixtures in comparison to aqueous media), electrophoretic mobility, and give more opportunities to control the overall separation processes, manipulate selectivity and to achieve separations unworkable in aqueous buffer [39,40,41]. During the optimization of CE conditions, it should be taken into consideration that the pKa values of acids and bases may be totally different in aqueous and nonaqueous media due to differences in dielectric constant between solvents, which also impacts on the mobilities of divalent ionic species, absorbance to the wall of the capillary, and finally affects the electroosmotic flow [42]. Apart from the effects of organic solvents on the acid-base properties of analyzed compounds, ion–ion interactions resulting from the presence of buffer electrolytes as well as other ionic modifiers in the background electrolyte and ion-solvent interactions could considerably impact the analyte’s electrophoretic mobility [41]. An unwanted phenomenon of poor reproducibility in migration time occurs when analyte absorption onto the capillary wall changes its conditions and, as a result, affects the magnitude of electroosmotic flow. This happens especially when a bare fused silica capillary is employed for analysis and it is little wonder that the interaction between samples and the inner capillary’s wall affects peak shape, resolution, and efficiency. This light capillary coating and surface modification establishes a good direction for future research and development of the technique [43].

#### 2.2.7. Field of Application

Methods based on capillary zone electrophoresis may be successfully employed, even for a full-scale quality analysis of herbal formulations, as was proved in the study reported by Xu et al. In this study, a comprehensive, rapid, and accomplishable electrophoretic method for the simultaneous separation and determination of seven constituents in Guan–Xin–Ning injection was elaborated and subsequently employed for quality control purposes [44]. 

CZE was successfully employed for the quantification of different classes of secondary metabolites in plant extracts among others: phenolic compounds, coumarins, protoberberines, curcuminoids, inorganic cations, isoquinoline alkaloids, iridoid glycosides, benzoic acid compounds quinolizidine alkaloids, and triterpene acids. This technique was also used for the determination of various active constituents and adulterants in herbal formulations. This kind of utilization is extremely important for the quality control of herbal medicinal preparations (for details see Table 1). A detailed analysis of the column entitled “Remarks” confirms the abovementioned ways to increase separation performance and detection sensitivity of capillary zone electrophoresis.

**Table 1:** Application of capillary zone electrophoresis.

Sample	Analytes	BGE	Detection	LOQ (µg mL^−1^)	Remarks	Ref.
“samgiumgagambang” (SGMX)	5–hydroxymethyl-furaldehyde, geniposidic acid, chlorogenic acid, paeoniflorin, 20–hydroxyecdysone, coptisine, berberine, luteolin and glycyrrhizic acid	70 mM borate buffer containing 10% methanol (pH 9.5)	UV (230 nm)	5.0–100.0	no significant difference between HPLC and CE results	[10]
12 herbal preparations used for the treatment of diabetes	metformin, chlorpropamide, glibenclamide and gliclazide	sodium acetate 20 mM L^−1^ (pH 10.0)	CM	3.21, 2.01, 4.46 and 5.77	determination of hypoglycemics as adulterants	[7]
26 herbal formulations	furosemide, hydrochlorothiazide, chlorthalidone, amiloride, phenolphthalein, amfepramone, fluoxetine and paroxetine	phosphate buffer (pH 9.2)	CM	5.14–11.01 mg/kg	determination of adulterants in herbal formulations for weight loss	[9]
7 *Aloe* plant species, 10 *Aloe* pharmaceutical preparations	aloin A and B	20.0 mM borate buffer with 50 mM SDS and 10 mM β–CD (pH 9.3)	LIF	0.025	microchip capillary electrophoresis (MCE)	[22]
*Abelia triflora* extract	scutellarein and caffeic acid	40 mM borax buffer (pH 9.2)	UV (200 nm)	2.5		[45]
*Aconite radix*	aconitine, mesaconitine, hypaconitine, benzoylaconine, benzoylmesaconine and benzoylhypaconine	200 mM Tris, 150 mM perchloric acid and 40% 1,4–dioxane (pH 7.8)	UV (214 nm)	0.14, 0.13, 0.14, 0.14, 0.13 and 0.15	LOD/LOQ ng mL^−1^	[24]
*Aconitum carmichaeli* (*Aconiti radix*: Chinese name: chuanwu)	aconitine, mesaconitine and hypaconitine	25 mM borax– 20 mM 1–ethyl–3–methylimidazo–lium tetrafluoroborate (pH 9.15)	ECL	5.62 × 10^−8^, 2.78 × 10^−8^, 3.50 × 10^−9^ mol L^−1^ 0.036, 0.018 and 0.002	LOD/LOQ ng mL^−1^	[46]
*Aesculus hippocastanum* (dry, hydro-alcoholic and hydroglycolic extracts)	β–escin	25 mmol L^−1^ bicarbonate–carbonate buffer (pH 10.3)	UV (226 nm)	38760		[47]
*Aesculus hippocastanum L.*, *Cichorium intybus L.*, *Melilotus officinalis L.* and *Juniperus communis L. “Pendula”*	aesculin, aesculetin, umbelliferone, dihydrocoumarin	20 mM borax in 5% methanol (pH 10.1)	UV (194 and 206 nm)	0.4–2.5 ppm		[48]
*Areca* nut	arecoline (methyl–1,2,5,6–tetrahydro–1–methylnicotinate)	20 mmol L^−1^ phosphate with 10 mmol L^−1^ BMImBF_4_ buffer (pH 7.50)	ECL	0.00077	LOD/LOQ pg mL^−1^	[23]
*Belamcandae rhizoma*	tectoridin and irigenin	borate buffer (pH 9.8)	AM	nd, LOD: 0.111 and 0.076	detection electrode based on the composite of carbon nanotubes and polylactic acid	[27]
*Bergeniae rhizoma*	arbutin and bergenin	50 mM borate buffer (pH 9.2)	AM	0.057 and 0.076	carbon nanotube–epoxy composite electrode	[28]
*Cacumen platycladi*	rutin, quercitrin, kaempferol and quercetin	50 mM sodium borate buffer (pH 9.2)	AM	0.110, 0.085, 0.063, 0.070	a fabricated graphene/poly(ethylene–co–vinyl acetate) composite electrode	[29]
*Camptotheca acuminata* (Camptotheca bark and fruit)	camptothecin alkaloids (CPT, 9–ACPT 9–MCPT HCPT, 7–EHCPT)	25 mM borate buffer containing 20 mM Sulfobutylether–β–CD and 20 mM ionic liquid [EMIM] [L–Lac] (pH 9.0)	UV (254 nm)	0.00020–0.00078	Large-volume sample stacking	[49]
*Cassia tora* (*Cassiae semen* and Cassia seed tea)	physcion, aloe–emodin, chrysophanol, emodin, aurantio–obtusin, rhein	10 mM Na_2_HPO_4_ and 6 mM Na_3_PO_4_ 15% methanol (*v*/*v*) (pH 11.8)	UV (254 nm)	1.11–4.67	an accelerated solvent extraction procedure	[50]
*Catha edulis*	cathinone, cathine, and phenylpropanolamine	25 mM TRIS phosphate buffer (pH 2.5)	UV (210 nm)	0.4		[51]
Chamomile and linden flower extracts	apigetrin, naringin, naringenin, catechin, galangin, apigenin, luteolin, quercetin, myricetin, kaempferol and kaempferide	40 mM borate buffer (pH 8.9)	UV (210 nm)	0.252–2.142		[52]
*Chelidonium majus* L	protopine, chelidonine, coptisine, sanguinarine, allocryptopine, chelerythrine, and stylopine	20 mM phosphate buffer (pH 3.1)	UV–LEDIF	0.06–5.5		[53]
*Chuanxiong rhizoma* (*Ligusticum wallichii*)	vanillin, ferulic acid, vanillic acid, caffeic acid and protocatechuic acid	50 mM borate buffer (pH 9.2)	AM	nd	carbon nanotube (CNT)–polydimethylsiloxane (PDMS) composite electrode	[54]
*Combretum aculeatum* extracts	punicalagin	25 mM, phosphate buffer (pH 7.4)	UV (280 nm)	60 ppm		[55]
*Connarus perrottetii* var. *angustifolius* (aqueous infusions, ethanolic extracts and butanolic extracts)	catechin and rutin	20 mM borate buffer containing 15% methanol (*v*/*v*), (pH 9.2)	UV (230 nm)	0.97 and 2.46		[56]
*Coptidis rhizoma* and berberine hydrochloride tablets	berberine	10 mM L^−1^ PBS (pH 7.81)	ECL	0.005	LOD/LOQ ng mL^−1^	[34]
*Coreopsis tinctoria* Nutt.	taxifolin–7–*O*–glucoside, flavanomarein, quercetagetin–7–*O*–glucoside, okanin 4′–*O*–glucoside, okanin and chlorogenic acid	50 mM borate buffer containing 15% acetonitrile (pH 9.0)	UV (280)	2.34–12.94		[57]
Daturae flos	atropine, scopolamine, and anisodamine	40 mM phosphate buffer containing 20% *v*/*v* methanol and 30% *v*/*v* acetonitrile (pH 7.0)	UV (196 nm)	0.50 (LOD)	capillary coated by graphene oxide	[58]
Duyiwei capsule and dried crude drug of *Lamiophlomis rotata*	8–*O*–acetylshanzhiside methylester and 8–deoxyshanzhiside, apigenin, quercetin and luteolin	10 mM sodium tetraborate–20 mM NaH_2_PO_4_–15% (*v*/*v*)methanol (pH 8.5)	UV (238 nm)	nd, nd, LOD: 2.6–9.2		[59]
*Echium vulgare* L. and *Echium* *russicum* L. *radix*	shikonin and rosmarinic acid	50 mM borate buffer (pH 9.5)	UV (218 and 202 nm)	nd, LOD: 0.603 and 0.270 ppm		[60]
*Ephedra sinica herba*	ephedrine and pseudoephedrine	80 mM of NaH_2_PO_4_, 15 mM of β–CD and 0.3% of hydroxypropyl methyl–cellulose (pH 3.0)	UV (214 nm)	nd, LOD: 0.7 and 0.6	Field-Amplified Sample Injection	[31]
*Epilobium parviflorum* extracts	caffeic acid, cinnamic acid, p–coumaric acid, ferulic acid, protocatechuic acid, syringic acid and vanilic acid	200 mM borate buffer with 37.5% methanol, 0.001% hexadimethrine bromide, and 15 mM 2–hydroxypropyl–β–CD (pH 9.2)	UV (214 nm)	0.032–0.094	On-line transient isotachophoretic preconcentration	[20]
*Epimedii herba* (Yin–Yang–Huo)	epimedin C, icariin, diphylloside A, epimedoside A and icarisoside A	30 mM borate buffer containing 40% methanol (pH 9.5)	UV (270 nm)	3.0, 2.0, 4.0, 2.0 and 3.0	coupled with SPE	[61]
Fengshi Maqian tablets and Yaotongning capsules	strychnine and brucine	75 mM phosphate buffer with 30% methanol (*v*/*v*) (pH 2.5)	UV (203 nm)	0.01	sample preconcentration method by two-step stacking	[62]
*Forsythia suspensa*	galacturonic acid and glucuronic acid	130 mM sodium hydroxide, 36 mM disodium hydrogen phosphate dihydrate and 0.5 mM cetyltrimethylammonium bromide (pH 12.28)	UV (270 nm)	10.68 and 12.64	reversed electroosmotic flow (EOF) to improve separation of neutral sugars	[63]
*Forsythia suspensa fructus* and commercial extracts	phillyrin, phillygenin, epipinoresinol–4–*O*–β–glucoside, pinoresinol–4–*O*–β–glucoside, lariciresinol, pinoresinol, isolariciresinol and vladinol D	40 mM borate buffer containing 2 mM β–CD and 5% methanol (*v*/*v*) (pH 10.30)	UV (234 nm)	3.00–4.38		[21]
*Forsythiae suspensae fructus*	oleanolic acid, ursolic acid and betulinic acid	50.0 mM L^−1^ borax and 0.5 mM L^−1^ β–cyclodextrin (β–CD) (pH 9.5)	UV (200 nm)	4.8, 4.6 and 5.9		[30]
*Fritillariae Thunbergii bulbus* (chloroform extracts)	peimine and peiminine	66% MeOH–ACN (1:1, *v*/*v*), 34% aqueous buffer containing 15 mM NaH_2_PO_4_, 2.5 mM NED, 4 mM H_3_PO_4_ (pH 3.0)	UV (214 nm)	nd., LOD: 3.9 and 4.1	NED as the UV absorbing probe	[64]
*Garcinia cambogia* (fruit rinds) and *Hibiscus sabdariffa* (calyx)	sodium salts of (1S,2R)–hydroxycitric and (1S,2S)–hydroxycitric acids	50 mM sodium phosphate buffer (pH 7.0)	UV (193 nm)	32.89–68.52		[65]
*Geranii herba*	rutin, hyperin, kaempferol, corilagin, geraniin, gallic acid, and protocatechuic acid	50 mM borate buffer (pH 9.2)	AM	nd, LOD: 30.9–682.8	graphene/poly(methyl methacrylate) composite electrode as a sensitive amperometric detector	[66]
*Ginkgo biloba* extract and rutin tablet,	epicatechin, rutin, and quercetin	10.0 mM borate and 0.5 mM luminol (pH 8.5)	CL	6 × 10^−7^, 5 × 10^−7^ and 1 × 10^−6^	ultrasensitive determination	[67]
*Glycyrrhiza uralensis* Fisch *radix*	glycyrrhetinic acid and glycyrrhizic acid	10 mM borate buffer (pH 8.8)	UV (268 nm)	6.2 and 6.9	On-line extraction coupled with flow injection and CE	[68]
Guan–Xin–Ning (GXN) injection	caffeic acid, danshensu, ferulic acid, isoferulic acid, salvianolic acid A, salvianolic acid B, tertamethylpyrazine	35 mM SDS and 45 mM borate solution (pH 9.3)	UV (212 nm)	1.5–4.90		[44]
*Hippophae rhamnoides* extract and Cerutin^®^ tablets	quercetin and rutin	40 mM L^−1^ borax (pH 9.2)	EC	0.475 and 0.726	hot platinum microelectrodes, flow injection analysis	[33]
*Houttuyniae herba*	rutin, isoquercitrin, quercitrin, and chlorogenic	50 mM borate buffer (pH 9.2)	AM	41.4, 31.8, 38.2 and 65.6	graphene/polystyrene composite electrode for amperometric detection	[69]
*Hypericum perforatum* and *Hypericum annulatum*	chlorogenic acid, epicatechin, hyperoside, rutin, quercitrin and quercetin	40 mM borate buffer, 50 mM SDS and 12% acetonitrile	UV (348, 208, 370, 370 and 318)	4.960–9.458 ppm	Non-significant differences between CE and HPLC	[35]
*Isatidis radix*	benzoic acid, salicylic acid and ortho–aminobenzoic acid	20 mM borate and 30 mM sodium dodecyl sulfate buffer containing 2 mMb–CD and 4%methanol (*v*/*v*), (pH 9.8)	UV (250 nm)	nd, LOD–800		[70]
Komplex Kurkumin^®^ (curcumin 375 mg, demethoxycurcumin 100 mg and bisdemethoxycurcumin 25 mg)	curcumin, demethoxycurcumin and bisdemethoxycurcumin	50 mM/ L CAPS, 100 mg mL^−1^ of HP–β–CD and2 gL−1 of HEC	UV–VIS (480 nm)	5.30, 4.57 and 6.20	unconventional hydrodynamically closed CE systems	[71]
*Lam– iophlomis* *rotate and Cistanche*	homovanillyl alcohol, hydroxytyrosol, 3,4–dimethoxycinnamic acid, and caffeic acid	50 mM borate–100 mM phosphate buffer in addition to 5.0 mM L^−1^ β–CD (pH 9.48)	UV (290 nm)	nd, LOD: 0.0051–0.029		[72]
*Lycoridis radiatae bulbus*	galanthamine	18 mmol L^−1^ phosphate buffer (pH 9.0)	ECL	nd, LOD: 0.00025		[73]
*Lycoris radiata*	galanthamine, homolycorine, lycorenine and tazetteine	10.0 mmol L^−1^ PBS (pH 8.0)	ECL	nd, LOD: 0.014, 0.011, 0.0018 and 0.0031	Ultrasonic-assisted extraction	[74]
*Lysium chinensis folium*	mannitol, sucrose, glucose, and fructose	50 mM NaOH	AM	0.120, 0.394, 0.126 and 0.155	Far-infrared-assisted extraction	[75]
*Macleaya cordata* and *Chelidonium majus* extracts	chelerythrine and sanguinarine	40 mM ammonium acetate–acetic acid–water buffer containing 50% (*v*/*v*) formamide (pH 2.90)	LIF	nd, LOD: 5.0 and 0.002	microchip electrophoresis	[76]
*Magnolia officinalis* and Huoxiang Zhengqi Liquid.	honokiol and magnolol	16 mmol L^−1^ sodium tetraborate, 11% methanol (pH 10.0)	UV (210 nm)	1670 and 830		[77]
*Origanum vulgare* and *Romanian propolis*	resveratrol, pinostrobin, acacetin, chrysin, rutin, naringenin, isoquercitrin, umbelliferone, cinnamic acid, chlorogenic acid, galangin, sinapic acid, syringic acid, ferulic acid, kaempferol, luteolin, coumaric acid, quercetin, rosmarinic acid and caffeic acid	45 mM borate buffer with 0.9 mM sodium dodecyl sulfate (pH = 9.35)	UV (280 nm)	0.07–5.77		[78]
*Orthosiphon stamineus* Benth.	rutin, carnosolic acid, caffeic acid, rosmarinic acid, quercetin, luteolin, apigenin and cinnamnic acid	50 mM borate buffer (pH 9.0)	UV (200 nm)	0.053, 0.053, 0.046, 0.040, 0.040, 0.030, 0.023 and 0.020	large volume sample stacking with polarity switching	[32]
*Peganum harmala semen* infusions	harmine, harmaline, harmol, harmalol, harmane, and norharmane	50 mM tris–HCl (pH 7.8) with 20% (*v*/*v*) of methanol	UV (254 nm)	0.1–8.3		[79]
*Penicillium glaucum*, *P. tenuifolium*, *P. dubium and P. fugax* fruits	morphine, codeine and thebaine	100 mM sodium phosphate buffer, containing 5 mM α–CD (pH 3.0)	UV (214)	2.0	Ultrasound-assisted extraction	[80]
*Phellodendri* *chinensis cortex*	berberine, palmatine and jatrorrhizine	20 mM phosphate buffer with methanol 10% (*v*/*v*), (pH 7.0)	UV	0.3	imprinted solid-phase microextraction	[81]
Pholia magra (*Cordia ecalyculata vell*, 500 mg/capsule), *Persea americana* and *Cyperus rotundus*	NH_4_^+^, K^+^, Ca^2+^,Na^+^, Mg^2+^, Mn^2+^, Tl^3+^, Cr^3+^, Pb^2+^, Cd^2+^, Zn^2+^,Cu^2+^, Co^2+^, and Ni^2+^	30 mM 2–N–MES /histidine, 1.5 mM 18–crown–6 ether, and 1 mM citric acid (pH 6.0)	C^4^D	0.093, 0.182, 0.405, 0.475, 0.077, 0.170, 1.478, 0.988, 2.008, 1.749, 0.454, 1.193, 0.817 and 0.632		[82]
*Phyllanthus urinaria*	rutin, quercetin, ferulic acid, caffeic acid, and gallic acid	10 mM borate buffer (pH 9.0)	AM	nd, LOD–3.36, 0.45, 0.097, 0.072 and 1.00		[83]
*Plumula nelumbinis*	neferine, liensinine, isoliensinine, rutin and hyperoside	50 mM borate buffer (pH 9.2)	AM	0.42, 0.31, 0.38, 0.35 and 0.39	far infrared-assisted solvent removal	[84]
*Portulaca oleracea* L., *Crataegus pinnatifida* and *Aloe vera* L.	linolenic acid, lauric acid, p–coumaric acid, ascorbic acid, benzoic acid, caffeic acid, succinic acid, and fumaric acid	40 mM H_3_BO_3_–40 mM Na_2_B_4_O_7_ (pH 8.70)	UV (200 nm)	nd, LOD: 0.02–3.44	field enhancement sample stacking for	[85]
propolis	pinocembrine; ferulic acid; p–coumaric acid; quercetin; and caffeic acid	100 mM borate buffer (pH = 8.7)	EC	nd, LOD: 0.1–0.5		[13]
*Puerariae radix*	3′-methoxypuerarin, puerarin, 3′-hydroxypuerarin, ononin, daidzin, daid–zein and genistin	35 mM sodium tetraborate, 9.0 mM sulfobutylether-β-CD α-cyclodextrin (pH9.34)	UV (254 nm)	2.5–9.5		[86]
Reduning injection	caffeic acid, isochlorogenic acid A, isochlorogenic acid B, isochlorogenic acid C, chlorogenic acid, neochlorogenic acid and cryptochlorogenic acid	20 mM NaH_2_PO_4_, 10 mM β–CD and 5% ACN (pH 4.2)	UV (325 nm)	0.8–1.5	DPPH–CE–DAD	[87]
*Rhodiola*	salidroside and tyrosol	50 mM borate buffer (pH 9.8)	AM	LOD: 0.72 and 0.39	a novel graphene/poly(urea– formaldehyde) composite modified electrode as a sensitive amperometric detector	[88]
*Rourea minor* stems	bergenin derivatives and catechins (new natural products)	30 mM borax solution with (pH 10.5)	UV (205 nm)	6.2–18.8		[89]
*Salvia miltiorrhiza*, *S. przewalskii*, *S. castanea* and Danshen	protocatechuic aldehyde, salvianolic acid C, rosmarinic acid, salvianolic acid A, danshensu, salvianolic acid B and protocatechuic acid	20 mM sodium tetraborate (pH 9.0)	UV (280 nm)	0.47–1.19		[90]
Sappan Lignum (the dried heartwood of *Caesalpinia sappan L*,methanolic extract)	brazilin and protosappanin B	20 mM borate buffer containing 6% *v*/*v* of methanol (pH 9.2)	UV (254 nm)	0.28 and 0.15	online concentration with acid barrage stacking	[91]
*Scutellariae barbata* extract	baicalein, baicalin, and quercetin	0.1 M borate buffer (pH 9.0)	EC	< 0.22		[92]
Shuxuening Injection	clitorin, rutin, isoquercitrin, quercetin–3–*O*–d–glucosyl]–(1–2)–l–rhamnoside, kaempferol–3–*O*–rutinoside, kaempferol–7–*O*–β–d–glucopyranoside, apigenin–7–*O*–Glucoside, quercetin–3–*O*–[2–*O*–(6–*O*–p–hydroxyl–E–coumaroyl)–d–glucosyl]–(1–2)–l–rhamnoside, 3–*O*–{2–*O*–[6–*O*–(p–hydroxyl–E–coumaroyl)–glucosyl]}–(1–2) rhamnosyl kaempfero	20 mM phosphate 5 mM β–cyclodextrin (β–CD), 40 mM sodium dodecyl sulfate and 7.5% ACN (pH 7.0)	UV–VIS (360 and 405 nm)	0.04–0.09	On-line 2,2′–Azinobis–(3–ethylbenzthiazoline–6–sulphonate)–ccapillary electrophoresis–diode array detector	[93]
*Sophora flavescens*	cytisine, sophocarpine, matrine, sophoridine, and oxymatrine	110 mM monosodium phosphate isopropanol (85:15, *v*/*v*) (pH 3.0)	UV (214 nm)	nd, LOD: 0.0004–0.0013	subcritical water extraction and field amplified sample stacking	[94]
*Sophora flavescens* (extract from the dried root)	matrine, oxymatrine, and sophoridine	50 mM sodium tetraborate solution, 500 mM boric acid and 1.2 mM citric acid (pH 7.98)	UV (210 nm)	60–100		[36]
*Swertia mussotii* Franch and preparations (herbs, granular, capsules)	oleanolic acid, ursolic acid, quercetin, and apigenin	50 mM borate–phosphate buffer with 5.0 × 10^−3^ mol L^−1^ β–cyclodextrin (pH 9.5)	UV (250 nm)	0.6829, 0.4007, 0.0124 and 0.5076		[95]
thyme and parsley extracts	luteolin and apigenin	20 mM borate buffer and methanol (90: 10, *v*/*v*), (pH 10.0)	UV (210 nm)	2.98 and 1.41		[96]
traditional Chinese medicines, *Hippophae rhamnoides*, *Hypericum perforatum*, and *Cacumen platycladi*	rutin, quercetrin, quercetin, kaempferol, kaempferide, catechin, apigenin and luteolin	18 mM borate buffer (pH 10.2)	AM	0.28, 0.22, 0.26, 0.24, 0.24, 0.22, 0.15 and 0.17		[97]
*Trichilia catigua*	epicatechin and procyanidin B2	80 mM borate buffer with 2–hydroxypropyl–β–cyclodextrin 10 mmol L^−1^, (pH 8.80)	UV (214 nm)	17.16 and 15.26	CE method faster, more efficient, less expensive, less polluting than previously developed HPLC method	[98]
*Trifolium alexandrinum* seed	soyasaponin I, azukisaponin V, bersimoside I and bersimoside	80 mM borate buffer containing 24 mM β–CD (pH 10)	UV (195 nm)	23.33, 21.64, 23.30 and 22.94	diastereomeric separation in	[99]
*Urceola rosea* leaf extracts	five phenolic compounds	25 mM sodium tetraborate decahydrate solution with (pH 8.5)	UV (254 nm)	10.9–20.8	CE method was well comparable to HPLC	[100]
*Valeriana officinalis* extracts	acacetin, diosmetin, chlorogenic acid, kaempferol, apienin, luteolin, p–hydrox–benzoic acid and caffeic acid	60 mM borate buffer (pH 9.2)	AM	0.033–0.4		[101]
Yansuan Xiaobojian Pian (berberine tablets), and plant samples: Goldthread, Amor Cork Tree, Goldenseal, Plantain, Tree Tumeric, Yellow Root, Bupleurum and Oregon Grape	berberine	20 mM acetic acid, 35 mM 2–HP–β–CD, and 20% methanol (pH 5.0)	LIF	nd, LOD: 0.016		[102]
Yinqiaojiedu tablet	liquiritin, chlorogenic acid, and glycyrrhizic acid	103.1 mM boric acid, 51.6 mM sodium borate, 9.8 mM disodium hydrogen phosphate, and 15.6 mM sodium dihydrogen phosphate (pH 7.86)	UV (254 nm)	0.41, 0.79 and 0.68		[103]

nd—no data, LOQ—limit of quantification, LOD—limit of detection, CM—conductometric, β—CD–β–cyclodextrin, LIF—Laser Induced Fluorescence, Tris—tris(hydroxymethyl)aminomethane, ECL—electrochemiluminescence, AM—amperometric, UV—LEDIF–ultraviolet light-emitting diode-induced native fluorescence, PBS—sodium phosphate buffer solution, NED—N-(1–naphthyl)ethylenediamine dihydrochloride, SDS—sodium dodecyl sulfate, CL—chemiluminescence, SPE—solid phase extraction, MES—morpholinoethanesulfonic acid, ACN—acetonitrile, capacitively coupled, C^4^D—contactless conductivity, DPPH—1,1-diphenyl-2-picryl-hydrazyl, DAD—diode array detector, 2-HP-β-CD-(2–hydroxypropyl)-β-cyclodextrin.

### 2.3. Micellar Electrokinetic Chromatography (MEKC) 

MEKC is a powerful electrophoresis-driven separation technique, which offers good selectivity, high efficiency, optimization flexibility, and significantly reduces organic solvent consumption during its operation. However, it is not possible to avoid organic solvent consumption when MEKC is applied to the analysis of medicinal plant materials or pharmaceutical formulations. This technique allows for the resolution of both neutral and charged compounds and may be applied for the analysis of a broad selection of active constituents; for instance, flavonoids in herbal raw material. The running buffer in MEKC is fortified with surfactants at a concentration exceeding their critical micelle concentration, that leads to forming micelles. The micelles arrange for a pseudostationary phase that enables the differential separating of analyzed compounds as a result of the influence of dispersed surfactants [101]. In reported studies, various pseudostationary phases were introduced. There are four major classes of surfactants: anionic, cationic, zwitterionic, and nonionic [104]. However, anionic surfactant, i.e., sodium dodecyl sulfate, was most frequently used in reported analyses. For more details see Table 2. 

In the past decade, MEKC was employed for the separation and quantification of different classes of secondary metabolites in plant extracts among others: coumarins, tanshinones, phenolic acids, terpenoids, iridoids, phenylethanoid glycosydes, phenylpropanoids, and flavonoids, saponin (see Table 2).

The compounds were detected and quantification was achieved mainly by UV absorption, but amperometric detection was also applied (see Table 2).

Recent studies confirm the high separation efficiency of MEKC and indicate tremendous potential for a wide range of analytical applications. Yang et al. employed polyvinylpyrrolidone-stabilized graphene-modified MEKC for the separation of tanshinones. The established method was successfully employed for the quality assessment of Danshentong capsules [105]. Cao et al. in turn used MEKC to resolve a mixture of flavonoids, phenolic acids, and saponin. In order to alter the electrophoretic behavior of analytes and to develop the resolution, they added ionic liquids-coated multi-walled carbon nanotubes to the running buffer, which influenced the partitioning of the analytes. Their results give real hope for the future analysis of complex samples based on considerable advantages in overcoming the effects of matrix-induced interferences exhibited in the study [106]. 

In the case of the use of large amounts of solvents, Chang and coworkers, in their paper, exhibited the elaboration of surfactant-assisted pressurized liquid extraction (PLE) for the effective extraction of flavonoids in *Costus speciosus* flowers prior to MEKC analysis. The reported work confirmed numerous advantages of PLE, i.e., short extraction time, simplicity, efficiency, automation, and environmental friendliness (organic-free). The PLC–MEKC approach enabled fast, eco-friendly, and effective extraction and assay of flavonoids in the abovementioned raw material [107].

In terms of improving the detection sensitivity in MEKC, a study by Chang et al. is reported, where the authors elaborated a magnetic iron oxide nanoparticle-based solid-phase extraction process in conjunction with the online concentration and separation of salicylic acid in in tobacco leaves through micellar electrokinetic chromatography–UV detection. The authors observed an approximately 1026-fold improvement in the detection sensitivity of the elaborated method in comparison to a single MEKC method without an online concentration [108].

Qualitative and quantitative methods based on MEKC are rapid, efficient, and eco–friendly, and are successfully employed for the routine quality control of herbal drugs and raw plant material (see Table 2).

**Table 2:** Application of micellar electrokinetic chromatography and microemulsion electrokinetic chromatography.

Sample	Analytes	BGE	Detection	LOQ (μg mL^−1^)	Remarks	Ref.
*Calendula officinalis*, *Hypericum perforatum*, *Galium verum* and *Origanum vulgare* extracts	(+)–catechin, (–)–epigallocatechin, (–)–epigallocatechin gallate, (–)–epicatechin gallate and (–)–epicatechin	10 mM KH_2_PO_4_ and 8.3 mM sodium tetraborate buffer with 66.7 mM SDS, (pH 7.0)	UV (210 nm)	0.010–0/047	LOD/LOQ ng mL^−1^	[109]
clove oil, litsea cubeba oil, and citronella oil	citronellal, citral (Z; E), a–pinene, limonene, linalool, and eugenol	20 m borate buffer, 50 mMSDS, 20% (v:v), (pH 9.5)	UV (210 nm)	0.8–5.9		[110]
*Costus speciosus flos* extract	rutin, quercitrin, and quercetin	10 mM phosphate, 10 mM borate, 50 mM SDS (pH 8.5)	UV (370 nm)	2.30, 1.57 and 1.07	surfactant–assisted pressurized liquid extraction	[107]
*Curcuma wenyujin* origin’s Chinese herbal medicines	curdine, curcumenol, germacrone, furanodiene, and β–elemene	1.3% SDS, 5.0% 1–butanol, 0.5% ethyl acetate and 10% acetonitrile in 10 mM borate buffer (pH 9.0)	UV (215 nm)	16.0–78.0	MSPD extraction coupled with MEEKC	[111]
Danshentong capsule (*Salvia miltiorrhiza*)	tanshinone IIB, dihydrotanshinone I, tanshinone I, cryptotanshinone, 1,2–dihydrotanshinone I, miltirone, and tanshinone IIA	10 mM borate buffer (pH 9.3) containing 30 mM SDS, 10% *v*/*v* 2–propanol and 6 µg mL^−1^ graphene	UV (260 nm)	8.73–19.10		[105]
*Hemidesmus indicus radix*	2–hydroxy–4–methoxybenzaldehyde, 2–hydroxy–4–methoxybenzoic acid, and 3–hydroxy–4–methoxybenzaldehyde	50 mM phosphate buffer with 65 mM of sodium taurodeoxycholate (pH 2.5)	UV (254 nm)	0.40, 2.5, and 0.7	MEKC results confirmed by HPLC–MS	[112]
*Heracleum sphondylium* herb and *Aesculus hippocastanum* cortex	coumarin, scoparone, isoscopoletin, esculin, esculetin, umbelliferone, xanthotoxin, byakangelicin, isopimpinellin, bergapten, phellopterin, xanthotoxol	50 mM sodium tetraborate, 45 mM SC, and 20% of methanol (*v*/*v*) (pH 9.00)	UV (214 nm)	1.70–4.772		[113]
*He–Shou–Wu*	hypohorine, THSG, epicatechin, proanthocyanidin B2, proantocyanidin B1, catechin and gallic acid	50 mM phosphate buffer containing 90 mM SDS and 2% (m/v) HP–β–CD (pH 2.5)	UV (210 nm)	<5.5	pressurized liquid extraction and short–end injection MEKC	[114]
*Larrea divaricata Cav.* extracts	nordihydroguaiaretic acid	20 mM phosphate buffer 10 mM SDS and 10% acetonitrile, (pH 7.5),	UV (283 nm)	1.06		[115]
Lianqiao Baidu pill	genistein, caffeic acid, glycyrrhizic acid ammonium salt, wogonoside	30 mmol L^−1^ SB, 95 mmol L^−1^ SDS, and 100 mmol L^−1^ boric acid (pH 9.30)	UV (214 nm)	0.77–1.85		[116]
*Ligaria cuneifolia* extracts	catechin, epicatechin, procyanidin B2, rutin, quercetin–3–*O*–glucoside, quercetin–3–*O*–xyloside, quercetin–3–*O*–rhamnoside, quercetin–3–*O*–arabinofuranoside, quercetin–3–*O*–arabinopyranoside and quercetin	20 mM borate buffer, 50 mM SDS mM β–CD and 2% *w*/*v* S–β–CD and 10% *v*/*v* methanol (pH 8.3)	UV (255 and 280 nm)	0.26– 1.33		[117]
*Lippia alba* leaves	genoposidic acid, 8–epi–loganin, mussaenoside, chrysoeriol–7–*O*–diglucuronide, triclin–7–*O*–diglucuronide, acetoside	50 mM borax buffer containing 75 mM SDS and 5% isopropanol		38.0–119.0	no statistically singificant differences beetween CE and HPLC	[118]
*Nicotiana tabacum* L. leaves	salicylic acid	TB buffer containing 100 mM SDS and 15% (*v*/*v*) acetonitrile (pH 10.0)	UV (205 nm)	nd, LOD: 0.0005	magnetic iron oxide nanoparticle–based solid–phase extraction procedure followed by an online concentration technique	[108]
*Petroselinum crispum*, *Rosmarinus officinalis*, *Thymus vulgaris L.*, *Origanum vulgare*, *Origanum majorana L.*, *Salvia officinalis L.*,*and Levisticum officinale*	apigenin	30 mmol L^−1^ sodium borate 10% acetonitrile, and 10 mmol L^−1^ sodium dodecyl sulfate (pH 10.2)	UV (390 nm)	0.28		[119]
*Plantago lanceolata*, *Plantago major*,*and Plantago asiatica* leaf extracts and biotechnological product, plant tissue cultures (calli) of *P. lanceolata.*	aucubin, catalpol, verbascoside and plantamajoside	15 mM sodium tetraborate, 20 mM TAPS and 250 mM DOC (pH 8.50)	UV (200 and 350 nm)	1360, 1630, 2350 and 2720		[120]
Qishenyiqi dropping pills	calycosin–7–*O*–β–d–glucoside, formononetin, dihydroquercetin, rosmarinic acid, danshensu, salvianolic acid B, protocatechuic acid, ginsenoside Rg_1_, ginsenoside Rb_1_	10 mM borate buffer (pH 9.0) containing 100 mM SDS, 6% propanol and 4 μg mL^−1^ ILs–MWNTs	UV (200 nm)	nd, LOD: 1.01–76.32	ionic liquids coated multi–walled carbon nanotubes as pseudo– stationary phase	[106]
*Salvia chionantha* and *Salvia kronenburgii* acetone extracts	horminone and 7–*O*–acetylhorminone	50 mM SDS, 25% metanol (pH:11.5)	UV (230 nm)	nd, LOD: 3.269 and 4.518		[121]
*Salvia miltiorrhiza*, *S. przewalskii*, and *S. castanea*	dihydrotanshinone I, cryptotanshinone, protocatechuic aldehyde, tanshinone I, tanshinone IIa, salvianolic acid C, rosmarinic acid, 9′–methyl lithospermate b, danshensu, salvianolic acid B and protocatechuic acid	15 mM sodium tetraborate with 10 mM SDS, 5 mM β–CD, 10 mM [bmim]BF_4_ and 15% ACN (*v*/*v*), (pH 9.8)	UV (254 nm)	0.90–4.63		[122]
Schisandra chinensis	schizandrin, schisandrol B, schisantherin B, schisantherin A, schisanhenol, deoxyschizandrin, schisandrin B	35 mM phosphate with 10 mM β–cyclodextrin (β–CD), 30 mM sodium dodecyl sulfate (SDS) and 10% ACN (pH 8.0)	UV (222 nm)	0.02–0.12	2,2–azinobis–(3–ethylbenzothiazoline–6–sulfonic acid)–sweeping micellar electrokinetic chromatography–diode array detector	[123]

nd—no data, LOQ—limit of quantification, LOD—limit of detection, SDS—sodium dodecyl sulfate, SB—sodium borate, MSPD—micro matrix solid phase dispersion, TAPS—N–[(1S,2S,3R)–2,3–bis(acetyloxy)–1–[(acetyloxy)methyl]heptadecyl]–acetamide, DOC—anionic detergents sodium deoxycholate, ILs–MWNTs—ionic liquids coated multi-walled carbon nanotubes, β–CD—β–cyclodextrin, HP–β–CD—hydroxypropyl–β–cyclodextrin, THSG–2,3,5,4′—tetrahydroxystilbene 2–*O*–β–d–glucoside, SC—sodium cholate.

### 2.4. Non-Aqueous Capillary Electrophoresis

Non-aqueous capillary electrophoresis (NACE) is a potent alternative to aqueous electrophoretic techniques, especially when it is difficult to separate lipophilic compounds. Separation of analytes is achieved using non-aqueous background electrolytes and the principle is based on the diverse physical and chemical properties of organic solvents. This great variety of solvents broadens the scope of separation selectivity. The low generation of current in nonaqueous media allows the use of high electric field strengths and wide bore capillaries and subsequentially allows a larger volume of the sample. Other advantages of NACE include better solubility of analytes in organic solvents, MS compatibility, and finally enhanced detection selectivity in many cases [124]. Analogically to the CZE sample, preconcentration techniques are applied in order to develop detection sensitivity in NACE. Field-amplified sample stacking, large-volume stacking using the electroosmotic flow pump, and transient isotachophoresis proved to be suitable for the abovementioned purpose and not only in aqueous CE. What is more, employment of LVSEP for NACE allowed the sensitive determination of organic anions at the nanomolar range using conventional UV detection and the introduction of ITP shortened the time of analysis [41]. 

The non-aqueous approach is not as prevalent as CZE or MEKC, but literature analysis indicates some applications for the analysis of herbal drugs and plant material. This technique was used in the study published by Hou et al. for the efficient separation and determination of five alkaloids in *Coptidis rhizoma*. In this work, surfactant-coated multi-walled carbon nanotubes provided a pseudostationary phase. Numerous parameters affecting NACE separation were studied, and in consequence, the authors noticed an important enhancement in the resolution due to the π–π interactions between the analyzed compounds and the surface of the carbon nanotubes in comparison to conventional NACE [125]. Meanwhile, Yuan et al. proposed a fast and uncomplicated method for the analysis of atropine, anisodamine, and scopolamine in *Deturae flos* extract by NACE coupled with electrochemiluminescence and electrochemistry dual detection. The running buffer was composed of acetonitrile and 2–propanol containing 1 M acetic acid, 20 mM sodium acetate, and 2.5 mM tetrabutylammonium perchlorate. Despite using a short capillary of 18 cm, the decoupler was not necessary and the separation performance was respectable [126]. Dresler et at. analyzed lipophilic compounds (hypericin and hyperforin) in *Hypericum* extracts with the non-aqueous capillary electrophoresis. The separation of the abovementioned constituents was conducted using bare fused silica 75 μm i.d. capillaries with an effective total length of 80.0 cm. The running buffer was a mixture of methanol, dimethylsulfoxide, and N–methyl formamide (3:2:1 *v*/*v*/*v*) as a solvent, with 50 mM ammonium acetate, 150 mM sodium acetate, and 0.02% (*w*/*v*) of cationic polymer hexadimethrine bromide to reverse the flow. At the same time, flavonoids and chlorogenic were evaluated with traditional CE as described above (Section 2.2). Only non-significant statistical differences were observed between the HPLC and CE results, namely the average differences between the particular metabolite ranged, e.g., from less than 10% for rutin and hypericin to ca. 1% for quercitrin [35]. The NACE method was also optimized for the simultaneous determination of major bioactive curcuminoids and some of the degradation products in turmeric milk and herbal commercial products. Non-aqueous BGE for separation of analytes was composed of sodium tetraborate, sodium hydroxide, methanol, and 1–propanol. Moreover, an innovative ultrasonication-assisted phase separation method was optimized and employed for extraction of the analytes in turmeric milk and subsequent direct injection of the extract into the capillary without any pretreatment [12]. The abovementioned NACE methods are simple, fast, convenient, and economical and applicable to analysis of herb extracts and commercial products (see Table 3).

**Table 3:** Application of nonaqueous capillary electrophoresis.

Sample	Analytes	BGE	Detection	LOQ (µg mL^−1^)	Remarks	Ref.
*Coptidis rhizoma*	coptisin, berberine, epiberberine, palmatine, jatrorrizine	20 mM sodium acetate in methanol–acetonitrile (80:20, *v*/*v*), 20% acetonitrile and 6 µg mL^−1^ SC–MWNTs	UV (254 nm)	0.31–0.34		[125]
*Daturae flos* extract	atropine, anisodamine, and scopolamine	acetonitrile and 2–propanol containing 1 M acetic acid, 20 mM sodium acetate and 2.5 mM tetrabutylammonium perchlorate	ECL and EC dual detection	0.5–50.0		[126]
*Hypericum perforatum* and *Hypericum annulatum*	hypericin and hyperfolin	methanol, dimethylsulfoxide, N–methyl formamide (3:2:1 *v*/*v*/*v*) with 50 mM ammonium acetate, 150 mM sodium acetate and 0.02% (*w*/*v*) of cationic polymer hexadimethrine bromide	UV (294 and 594)	2.191–2.948 ppm		[35]
Turmeric milk (*Curcuma longa*) and herbal products	curcumin, desmethoxycurcumin and bisdesmethoxycurcumin, vanillin, vanillic acid, ferulic acid, and 4–hydroxybenzaldehyde	a mixture of sodium tetraborate, sodium hydroxide, methanol and 1–propanol	UV–VIS (300 and 498 nm)	10.1–26.5	a novel ultrasonication–assisted phase separation method (US–PS) was used for extraction and subsequently the extract was directly injected into the capillary	[12]

nd—no data, LOQ—limit of quantification, LOD—limit of detection, SC-MWNTs—surfactant-coated multi-walled carbon nanotubes, ECL—electrochemiluminescence, EC—electrochemistry.

### 2.5. Capillary Electrochromatography (CEC)

Capillary electrochromatography is a hybrid technique because it merges features of both high performance liquid chromatography and capillary electrophoresis and may be applied for the determination of charged and neutral analytes. In CE, analytes are separated in a capillary column with electroosmotic flow as the driving force for bulk fluid movement. However, in capillary electrochromatography, the capillary contains a stationary phase as in HPLC. Hence, there is a capability to take advantage of different mechanisms to provide additional selectivity beyond that possible through HPLC or CE alone. This combination of CE has advantages, i.e., high-efficiency, low-solvent and sample consumption, and reverse-phase mechanism of HPLC makes this technique reliable and flexible and, what is more, it can be fully suitable for pharmaceutical analysis and can replace other more demanding techniques in terms of time and expenses [127,128]. On the other hand, in comparison to CZE, the optimization of CEC is more challenging, the efficiency is lower due to peak broadening and the reproducibility of retention times is poorer [129]. However, a continuous fulfilling CEC with nanoparticles as a pseudostationary phase coupled with MS detection demonstrates high separation efficiency, as well as high performance confirmed by such parameters as limit of detection, peak asymmetry, repeatability, and reproducibility [130]. Except for employing a detector with high sensitivity, other approaches to achieve good detection sensitivity, as well as resolution and separation efficiency, include bubble or Z-type cells to extend the optical path, and obviously sample preconcentration techniques. For instance, FASS and in-column detection [131,132]. CEC was reported for the fast separation and quantification of coumarins in *Angelica dahurica* extract. A methacrylate ester-based monolithic column was used as a stationary phase. In order to gain a significant raise in the selectivity, surfactant sodium desoxycholate was added to the mobile phase as the pseudostationary, so there was no need to increase the hydrophobicity of the stationary phase. The devised method was characterized not only by satisfactory separation and a running time of 6 min, but also by LOQs lower than 0.30 μg/mL^−1^ [133]. The second reported study describes a CEC method for the quality control of *Cnidii fructus* extracts. This method, taking advantage of the methacrylate ester-based monolithic column, was characterized by an acceptable resolution of LOQs between 1.0 and 2.8 μg/mL^−1^ and the time of operation was shortened to 5 min [134]. For more details see Table 4.

**Table 4:** Application of capillary electrochromatography.

Sample	Analytes	BGE	Detection	LOQ (µg mL^−1^)	Remarks	Ref.
*Angelica dahurica* extract	byakangelicin, oxypeucedanin hydrate, xanthotoxol, 5–hydroxy–8–methoxypsoralen and bergapten	30:70 *v*/*v* ACN–buffer containing 20 mM sodium dihydrogen phosphate (NaH_2_PO_4_) and 0.25 mM SDC (pH 2.51)	UV (210 nm)	<0.30	methacrylate ester–based monolithic column	[133]
*Cnidii fructus* extracts	isopimpinelline, bergapten, imperatorin and osthole	50% ACN and 50% of a 10 mM sodium dihydrogen phosphate (pH 4.95)	UV (210 nm)	1.0–2.8	poly(butyl methacrylate–co–ethylene dimethacrylate–co–[2–(metha-cryloyloxy)ethyl] trimethylammonium chloride) monolithic column	[134]

LOQ—limit of quantification, SDC—surfactant sodium desoxycholate, ACN—acetonitrile.

### 2.6. Capillary Electrophoresis–Mass Spectrometry (CE-MS)

Capillary electrophoresis has many advantages in HPLC (low solvent consumption, using inexpensive capillaries, short time of operation, high efficiency without sample retreatment) and can support complementary or supplementary information about the constitution of a sample. One of the limitations of CE techniques is the relatively poor sensitivity as a result of the injection of small sample volumes, which might be improved by the implementation of pre-concentration techniques. The other way is to take advantage of CE–MS hyphenation, which not only enhances LOD thanks to MS detection, but also allows for the measurement of the particular mass of analytes and offers structural information, including the opportunity to identify and determine co-migrating species in overlapping peaks [135]. In reported papers, authors have described the quantitative analysis mainly of alkaloid compounds in plant extracts/pharmaceutical formulations. 

In the study of Liu et al., the CEC–MS method, fully applicable for the quality evaluation of *Evodiae fructus*, was elaborated. It should be underlined that 4–16 fold improvement of detection limits was achieved in comparison to the CEC method with conventional UV detection [136]. Wang et al. proposed matrix solid-phase dispersion microextraction combined with CE in conjunction with quadrupole time-of-flight mass spectrometry for the quantification of three alkaloids in *Fritillariae Thunbergii bulbus*. It is noteworthy that in this method the reported LOQ value is in the ng mL^−1^ level [137]. All reported CE–MS methods were effectively employed for qualitative and quantitative analysis of bioactive components in plant extract and pharmaceutical preparations (see Table 5).

**Table 5:** Application of capillary electrophoresis with MS detection.

Sample	Analytes	BGE	Method	LOQ (µg mL^−1^)	Remarks	Ref.
*Catharanthus roseus*	vinblastine, vindoline, and catharanthine	20 mM ammonium acetate and 1.5% acetic acid	CE–MS	nd, LOD: 0.1–0.8		[135]
*Evodiae* fructus	limonin, evodiamine, and rutaecarpine	30% acetonitrile (ACN) in 1% ammonia aqueous solution	CEC–MS	3.1, 0.63 and 0.15	provided 4–16 folds improvement of LODs when compared with CEC–UV method	[136]
*Fritillariae Thunbergii bulbus*	peimine, peiminine, and peimisine	20 mM ammonium acetate with MS–grade water	CE–Q–TOF–MS	0.004–0.005	solid acids assisted matrix solid-phase dispersion micro-extraction	[137]
*Lycoris radiata* roots	lycorine, lycoramine, lycoremine, lycobetaine, and dihydrolycorine	ACN and methanol (1:2, *v*/*v*), which 40 mM ammonium acetate and 0.5% acetic acid	NACE ESI–IT–MS	0.04–0.24		[138]
*Psoralae fructus* and pharmaceutical preparations	bavachin and isobavachalcone	20 mM aqueous solution of ammonium acetate (pH 10.0)	CE–ESI–MS	nd, LOD: 0.06		[139]
*Banisteriopsis caapi*, *Datura stramonium*, *Mimosa tenuiflora*, *Peganum harmala*, *Voacanga africana*, Ayahuasca	harmaline, harmine, harmalol, norharmane, harmane, harmol, tetrahydroharmine, and tryptamine	58 mmol L^−1^ ammonium formate and 1.01 mol L^−1^ acetic acid in acetonitrile	NACE–MS	0.01, 0.01, 0.015, 0.012, 0.018, 0.019, 0.022 and 0.024		[140]
*Rheum* (Rhubarb, Dahuang) extracts	physcion, chrysophanol, and aloe–emodin	80% methanol and 20% acetonitrile with 20 mM ammonium acetate	NACE–ESI–MS/MS	nd, LOD:84, 180 and 210 ppb		[141]
*Stephaniae tetrandrae radix* and *Menispermum dauricum rhizoma*	tetrandrine, fangchinoline, and sinomenine	80 mM solution of ammonium acetate with mixture of 70% methanol, 20% ACN, and 10% water, which also contained 1% acetic acid	NACE–IT–MS	nd, LOD: 0.05, 0.08, and 0.15		[142]
*Tinosporae radix*	palmatine, cepharanthine, menisperine, magnoflorine, columbin and 20–hydroxy-ecdysone	methanol and acetonitrile (4:1; *v*/*v*), which contained 40 or 50 mM ammonium acetate and 0.5% acetic acid	NACE–ESI–MS	0.06–4.0		[2]

nd—no data, CE–Q–TOF–MS—capillary electrophoresis coupled with quadrupole time-of-flight mass spectrometry, ESI–IT–MS—electrospray ionization ion trap mass spectrometry, ESI–MS—electrospray ionization mass spectrometry, IT–MS—ion trap mass spectrometry.

## 3. Materials and Methods

The present literature review is based on PRISMA guidelines. The selection criteria for the articles for the review were formulated on the basis of the PICOS process (see Table 6). For the purpose of this review, articles from 2010 to 2019 were taken into consideration. Searching of the literature for this publication was performed between January 2021 and March 2021 using the PubMed and Web of Science databases. The search strategy took place with the use of the following keywords:

1. “capillary electrophoresis” AND
2. “pharmaceutical analysis” OR “determination” OR “quantification” AND
3. “herbal drugs” OR “medicinal plants” OR “plant extracts” OR “plant metabolites”.

In the PubMed database, a combination of terms ‘All fields’ and in Web of Science base terms ‘Topic’ was used, which searches titles, abstracts, author keywords, keywords, and more. Only articles in English, available full texts and articles delineating the quantitative analysis of bioactive components in medicinal plants and pharmaceutical formulations by CE are included in this review. The exclusion criteria were opinion letters, conferences, abstracts, and papers not written in English (for example, in Chinese). Publications restricted only to fingerprinting or separation without quantitative analysis were rejected. Additionally, articles with urine, human plasma and blood serum, and edible products such as the matrix were eliminated. Studies in which amino acids in plant tissues, enzyme inhibition or alternations of secondary metabolites in plants under different factors analyzed using CE were also not taken into account. Duplicates were removed and found articles were sorted by title, abstract, and then main text. The articles were excluded if they did not meet the inclusion criteria. Selection of appropriate works taking into account the inclusion and exclusion criteria were controlled by the three authors of this paper (M. G., A. P, M. K.) Selection of the publications by them was made on the basis of a qualitative and quantitative evaluation of articles from the PubMed and Web of Science databases, especially by title of paper, first name of the author, and year publication.

## 4. Conclusions

The present review summarized the state of the art applications of capillary electrophoresis over a past decade. The versatile application of CE-based methods was recorded due to the possibility of using different techniques of CE adapted to the substance to be determined and their numerous modifications. 

The present scrutiny reveals a large number of applications, including different formulations, various plant extracts, simultaneous identification, and quantification of even several active constituents in a complex matrix. In the reported works, CZE, MEKC, and NACE were successfully used for the assay of different classes of secondary metabolites, whereas NACE was employed for the analysis of lipophilic compounds, CEC for the analysis of coumarins and CE-MS mainly for alkaloid compounds. Due to its many advantages, such as little solvent and sample consumption, short time of operation, and high efficiency, CE is an attractive and eco-friendly approach in current pharmaceutical analysis and its continuous development gives hope for well-established, validated, and increasingly accurate and precise methods of quality control of pharmaceutical formulations and herbal raw material.

Among all reported methods, the most common is the CE–UV technique; however, in some cases, resolution and sensitivity are limited. For this reason, other methods of detection, such as conductometry, electrochemiluminescence, laser-induced fluorescence, and hyphenation of CE–MS, have successfully been applied. Moreover, among other electrophoretic techniques, MEKC and NACE are well established. Other ways to solve this problem are through the addition of some modifiers to the BGE, i.e., cyclodextrins are added as a chiral selectors during enantiomeric separation, or through introducing sophisticated extraction and/or pre-concentration techniques. The flexibility of CE is a great advantage, i.e., it includes many opportunities for optimizing the parameters of analysis, additives to BGE, introducing in-line and online preconcentration techniques and different methods of detection, and makes every electrophoretic technique capable of being used for the routine qualitative and quantitative analysis of active constituents in plant material or herbal formulation even at the ng mL^−1^ level. In some cases, a comparison of the results obtained with CE to HPLC methods exhibited no statistically significant differences. Moreover, differences in sensitivity are relevant only in the analysis of samples with very low analyte concentrations, which does not directly relate to pharmaceutical analysis, where the content of the active ingredient, for instance in tablets, is at the milligram level. This suggests the CE method may be better where it does not influence the quality of the analysis, because of its shorter time of execution, lower costs, and eco-friendly approach. It should also be noted that the future of CE is strongly connected to hyphenation with the MS technique because of its ability for both measuring molecular weight and for offering structural information. On the one hand, detection sensitivities of the reported methods based on CE-MS were relatively low, but in some cases, they were comparable to results achieved even with UV detection. On the other hand, more and more utilizations of CE-MS, as well as a constant development, indicate that this hyphenation is heading in the right direction. 

In conclusion, CE is a powerful analytical tool, and after adequate optimization, it could be an auspicious alternative to more expensive methods in the pharmaceutical quality control of herbal drugs and herbal raw material. 

## Figures and Tables

**Table 6:** PICOS (Population, Intervention, Comparison, Outcome, Study type).

	Inclusion Criteria	Exclusion Criteria
Population	herbal drugs and medicinal plants	garden and ornamental plants, vegetables and fruits, edible products, beverages
Intervention	use of CE method	other methods
Comparison	capillary electrophoresis vs. other methods	not applicable
Outcome	analysis of active constituents	different outcomes
Study type	original research articles, full articles, English language	review articles, reports, abstracts, articles with no quantitative information or details

## Data Availability

All data obtained during the research appears in the submitted article.
